# ND-FISH with New Oligo Probes for Chromosome Identification of *Cichorium intybus* Revealing Karyotypic Variation and Divergence of Asteraceae Species

**DOI:** 10.3390/plants13223135

**Published:** 2024-11-07

**Authors:** Meiling Chen, Chengzhi Jiang, Doudou Huang, Zhiqiang Zheng, Wenzhuo Yang, Guangrong Li, Chun Fu, Hong Liao, Wencong Long, Zujun Yang, Yaojun Yang

**Affiliations:** 1 Forestry and Bamboo Technology Innovation Industry Research Institute, Leshan Normal University, Leshan 614000, China; cmelia123@163.com (M.C.); fuchun421@aliyun.com (C.F.); 18608332688@163.com (H.L.); longwencong@163.com (W.L.); 2 School of Life Science and Technology, University of Electronic Science and Technology of China, Chengdu 610054, China; 202311140626@std.uestc.edu.cn (C.J.); 202221140532@std.uestc.edu.cn (D.H.); 202221140503@std.uestc.edu.cn (Z.Z.); 2022140902006@std.uestc.edu.cn (W.Y.); ligr28@uestc.edu.cn (G.L.); 3 Key Laboratory of Sichuan Province for Bamboo Pests Control and Resource Development, Leshan Normal University, Leshan 614000, China

**Keywords:** *Cichorium intybus* L., TRs, oligo, ND-FISH, chromosomes

## Abstract

Chicory (*Cichorium intybus* L., 2n = 18), belonging to the Asteraceae family, exhibits significant edible, medicinal, and pasture values. Moderate research has been performed on identifying Chicory species’ chromosomes using fluorescence in situ hybridization (FISH) and C-banding. Detailed karyotype comparisons with chromosome nomenclature have not yet been performed for Chicory and similar species. In this study, the tandem repeats (TRs) were predicted and mapped to chromosomal regions based on released *C. intybus* L. ASM2352571 genome assembly v1, and then compared to the genome of Lettuce (*Lactuca sativa* L.). Nine new oligo probes were then developed and employed for karyotypic investigation of endive, Lettuce, and Chicory mitotic metaphase using non-denaturing FISH (ND-FISH). By combining the conserved oligo probes for 5S rDNA and 18S rDNA with the unique ND-FISH signals of new TR-oligo probes, we can develop a high-resolution standard karyotype for the cultivars of Lettuce and Chicory. The occurrence of chromosome structure variations from the natural population of Chicory and Lettuce was also revealed by ND-FISH with multiple oligo probes. The current observation of the karyotype differences and divergences of *Lactuca* and *Cichorium* and the genomic research offers crucial information about the Asteraceae family’s genetic diversity, chromosomal dynamics, and evolutionary routes.

## 1. Introduction

Asteraceae is one of the largest plant families of angiosperms, containing more than 1620 genera and 24,000–35,000 species [1,2]. As the largest genus in the Asteraceae family, the Cichorieae tribe is morphologically well characterized by the presence of latex and perfect flowers within the capitulum [3]. Among members of this tribe, Cichorieae, Chicory (*Cichorium intybus*), Lettuce (*Lactuca sativa* L.), and endive (*Cichorium endivia*) are extensively distributed and under development as industrial plants due to their significant edible value, high-quality forage, and medicinal application [4,5,6]. Studies have concentrated on the therapeutic activities of the plant and the phytochemical compounds responsible for the medicinal reputation of *C. intybus* [7]. There is a lack of comprehensive molecular and cytogenetic reports concerning Chicory and its related species. For example, Rick et al. [8] conducted the cytogenetic evaluations for *C. endivia* and *C. intybus* using conventional staining and reported a diploid number of 2n = 18 for both species. Matoba et al. [9] performed a chromosomal study and observed the same primary chromosome number in Lettuce and its allied species. A study of the chromosome karyotype and C-banding analysis of Puna Chicory was conducted by Ge et al. [10]. Imani et al. [11] also identified eight ecotypes of *C. intybus* from Iran by chromosome counting. Because of the little variations in chromosomal lengths and insufficient C-bands, traditional cytological data are scarcely identifiable for the significantly lower genetic divergence among and between Cichorium and its related species.

Chromosome studies using FISH (fluorescent in situ hybridization) gradually replaced chromosome banding to become the most popular technique for chromosome identification in plants [12]. Bernardes et al. [4] observed intra- and interspecific karyotypic polymorphisms in cultivated *Cichorium* species revealed by using FISH and double fluorochrome staining with CMA (chromomycin A3) for GC-rich heterochromatic regions and DAPI (4′,6-diamidino-2-phenylindole) for AT-rich heterochromatic regions. However, the identified features representing FISH patterns provide evidence for the distribution of the primary chromosome markers. Revealing the high diversity of individual chromosomes remains challenging despite the apparent homogeneity of karyotypes. In FISH-based chromosome identification, repetitive DNA sequences are commonly utilized as probes [13]. The non-denaturing FISH (ND-FISH) technique, utilizing oligonucleotides specific to a repetitive DNA sequence, has been validated for its effectiveness in recognizing a specific chromosomal region or an entire chromosome through the use of computationally identified, synthesized in parallel, and fluorescently labeled probes [14]. The development of genome-wide TR oligos has significantly enhanced the resolution of karyotypes, thereby facilitating a more precise identification of chromosomal variants [15,16,17]. Recently, the genome sequencing of the Chicory [18] and its relatives, endive [18], Lettuce [19,20], and Stem Lettuce [2], has been completed, which enables the mining of representative TRs covering the whole genome for identifying all chromosomes among representative Asteraceae species.

The objective of this research is to (1) develop new oligo probes derived from genome sequences for FISH, enhancing the resolution of individual Chicory chromosome recognition for accurate karyotypic analysis on mitotic metaphase spreads; (2) investigate genetic variation within the Chicory population; and (3) provide insights into the genetic diversity and chromosome structure of various Asteraceae members, which will serve as germplasm for breeding cultivars of industrial Chicory products.

## 2. Results

### 2.1. Genomic Distribution of TRs in Chicory and Lettuce Genomes

The genome sequences of Chicory [19] and Lettuce [20] were obtained for the analysis of the proportion and distribution of tandem repeats (TRs) in each chromosome, utilizing TRF software (http://tandem.bu.edu/trf/trf.html (accessed on 10 May 2024)). As shown in Table 1, we obtained a total length of 84.4 Mb TRs in Chicory, which constituted 7% of the total 1.2 Gb assembled genome. The longest chromosome measured 161.7 Mb for 1C, while the lowest measured 98.2 Mb for 9C. The minimum percentage of chromosome sequence length attributed to TRs was 5.75% for chromosome 6C, while the maximum was 9.17% for chromosome 8C. The proportion of transposable elements in the 2.59 Gb Lettuce genome is merely 0.54%, measuring 14 Mb. The 1L chromosome contained the highest TR proportion at 1.58%, and the 3L chromosome had the lowest TR proportion at 0.19%. The coverage of TR content in nine homology groups exhibited abundant variation between Chicory and Lettuce genomes.

### 2.2. Chromosomal Location of rDNA Site and FISH Validation

**Figure 1:**
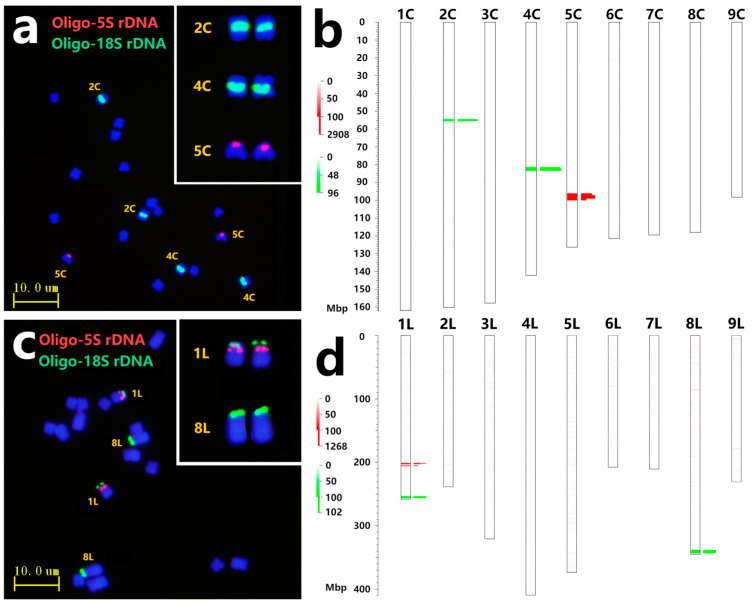
The ND-FISH results (**a**,**c**) and physical distribution (**b**,**d**) of Oligo-18S rDNA and Oligo-5S rDNA in chromosomes of Chicory (**a**,**b**) and Lettuce (**c**,**d**). ND-FISH for mitotic metaphase of Chicory (**a**) and Lettuce (**c**) showing the hybridization sites of Oligo-18S rDNA and Oligo-5S rDNA. Bars, 10 μm. The prediction of Oligo-18S rDNA and Oligo-5S rDNA in Chicory reference assembly ASM2352571v1 (**b**) and Lettuce T2T genome of the L. *sativa* cv. PKU06 (**d**) used default parameters for the blast and filter steps according to the website B2DSC. Red bars: the positions of Oligo-5S rDNA. Green bars: the positions of Oligo-18S rDNA.

Bernardes et al. [4] performed FISH analysis utilizing rDNA probes in Chicory, identifying 45S rDNA loci on chromosomes 1 and 3 and 5S rDNA loci on chromosome 8. Wang et al. [19] indicated that Lettuce possesses two nucleolus organizer regions (NORs) located on chromosomes 1 and 8. According to the prediction of tandem repeats in the Chicory genome, chromosomes 2C and 4C exhibited 18S rDNA loci close to centromeric regions, with 110 and 209 copies, respectively. Meanwhile, chromosome 5C showed abundant 5S rDNA sites around 96–99 Mb locations (Table S2, Figure 1b). ND-FISH with probes Oligo-18S rDNA + Oligo-5S rDNA was used to identify mitotic metaphase chromosomes in Chicory. Two pairs of chromosomes displayed distinct green signals in the centromeric regions. One pair, characterized by shorter chromosomes and a higher copy number, was designated chromosome 4C. The other pair, with longer chromosomes and a lower copy number, retained the name 2C. The probe Oligo-5S rDNA displayed strong red signals on a pair of chromosomes characterized as 5C chromosomes (Figure 1a). Likewise, the localization of rDNA sites in Lettuce indicated that chromosomes 1L and 8L carried 18S rDNA sites at the terminal region, and two additional 5S rDNA sites were located in the subterminal region of 1L near 18S rDNA sites (Table S1, Figure 1d). As shown in Figure 1c, the short arm of chromosome 1L contained strong Oligo-18S rDNA and Oligo-5S rDNA signals, while chromosome 8L contained only Oligo-18S rDNA signals. Furthermore, 5S rDNA sites were located on the short arm of 5C, indicating a reversed direction of the physical assembly location of Chicory. The predicted physical locations of tandem repeat using the B2DSC web server corresponded closely with the cytological positions, indicating the accuracy and reliability of the sequence assembly for Chicory and Lettuce in the rDNA sites.

### 2.3. ND-FISH of New TR Probes for Chromosome Identification in Cichorium intybus cv. Commander and Lettuce

**Figure 2:**
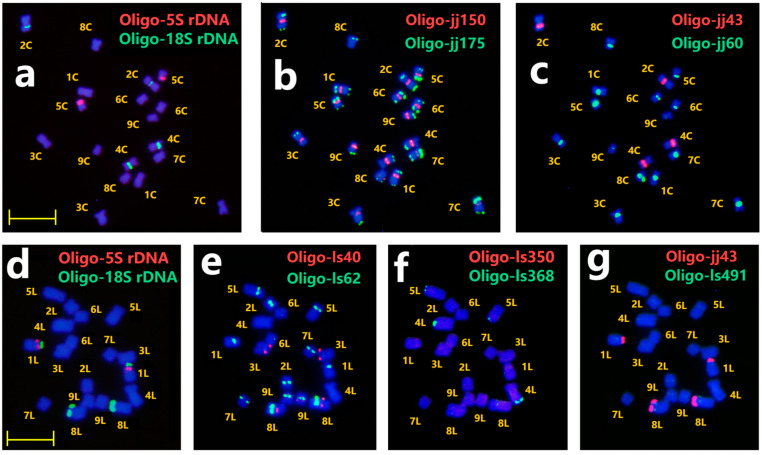
ND-FISH using multiple probes in Chicory (**a**–**c**) and Lettuce (**d**–**g**). The probes for FISH were Oligo-5S rDNA (red) + Oligo-18S rDNA (green) (**a**,**d**); Oligo-jj150 (red) + Oligo-jj175 (green) (**b**); Oligo-jj43 (red) + Oligo-jj60 (green) (**c**); Oligo-ls40 (red) + Oligo-ls62 (green) (**e**); Oligo-ls350 (red) + Oligo-ls368 (green) (**f**); Oligo-jj43 (red) + Oligo-ls491 (green) (**g**). Bars, 10 μm.

To establish the standard karyotype for Chicory and Lettuce, four and five new oligo probes for ND-FISH were developed using reference genome sequences of Chicory and Lettuce, respectively. The physical distributions and estimated copy numbers of nine oligo probes were determined (Figure S1). Figure 2a illustrates the rDNA signals, which were further analyzed using sequential ND-FISH with probes Oligo-jj150 + Oligo-jj175 in the second round of hybridization (Figure 2b). Probe Oligo-jj150 displayed weak hybridization signals in the centromeric regions of seven chromosome pairs, excluding 7C and 8C, as the predicted copy numbers corroborated. In the meantime, the probe Oligo-jj175 showed distinct hybridization signals in the telomeric or sub-telomeric regions of nearly all chromosomes, affecting one or both arms. Chromosomes 1C and 6C display two hybridization signals on their long and short arms. Chromosome 1C was longer than 6C. Chromosomes 3C and 9C demonstrated a single hybridization site located in the terminal region of the long arm. However, the 9C chromosome showed the shortest chromosome length. Furthermore, probe Oligo-jj43 and Oligo-18S rDNA had the same hybridization locus on 2C and 4C. However, the predicted copy numbers of probe Oligo-jj43 were even greater than probe Oligo-18S rDNA (Table S1, Figure S1). On the contrary, probe Oligo-jj60 displayed distinct signals on the centromeric region of seven pairs of chromosomes except 2C and 4C (Figure 2c). Further, probe Oligo-(AG)_10_ displayed slightly smaller hybridization signals on 8C chromosomes, and Oligo-Telo displayed weak signals on all nine chromosomes (Figure S2a,b). In Lettuce chromosomes, Oligo-ls62 showed distinct hybridization signals in the centromeric regions of seven chromosome pairs, excluding 3L and 4L. Moreover, distinct red signals from Oligo-ls40 were observed on chromosomes 3L, 8L, and 9L. Similarly, probe Oligo-ls368 exhibited clear telomeric signals on 4L and 5L, while probes Oligo-ls350 and Oligo-ls491 displayed weaker signals at 1L and 8L, respectively. According to the ND-FISH results, Oligo-jj150 and Oligo-ls62 serve as centromere-specific probes for Chicory and Lettuce, with their predicted centromere locations and copy numbers explained in Table S2. Therefore, in combination with chromosome length prediction and probes Oligo-5S rDNA + Oligo-18S rDNA, Oligo-jj150 + Oligojj175, and Oligo-jj43 + Oligojj60, the nine pairs of chromosomes in Chicory were recognizable. Eight probes (Oligo-5S rDNA, Oligo-18S rDNA, Oligo-ls40, Oligo-ls62, Oligo-ls350, Oligo-ls368, Oligo-ls491, and Oligo-jj43) were capable of characterizing all Lettuce chromosomes. Four Oligo probes (Oligo-jj150, Oligo-jj175, Oligo-jj43, and Oligo-jj60) and five probes (Oligo-ls62, Oligo-ls40, Oligo-ls350, Oligo-ls368, and Oligo-ls491) were designed based on reference Chicory and Lettuce genomes, respectively. Among these probes, Oligo-jj43 (Figure 2g) and Oligo-ls350 (Figure S2c,d) showed clear ND-FISH signals for both species, indicating their cross-species applicability, while the remaining seven probes demonstrated species-specific hybridization signals. The results indicate significant differences in genomic repetitive sequence distributions between Chicory and Lettuce.

### 2.4. Standard Karyotype of C. intybus cv. Commander and Lettuce

**Figure 3:**
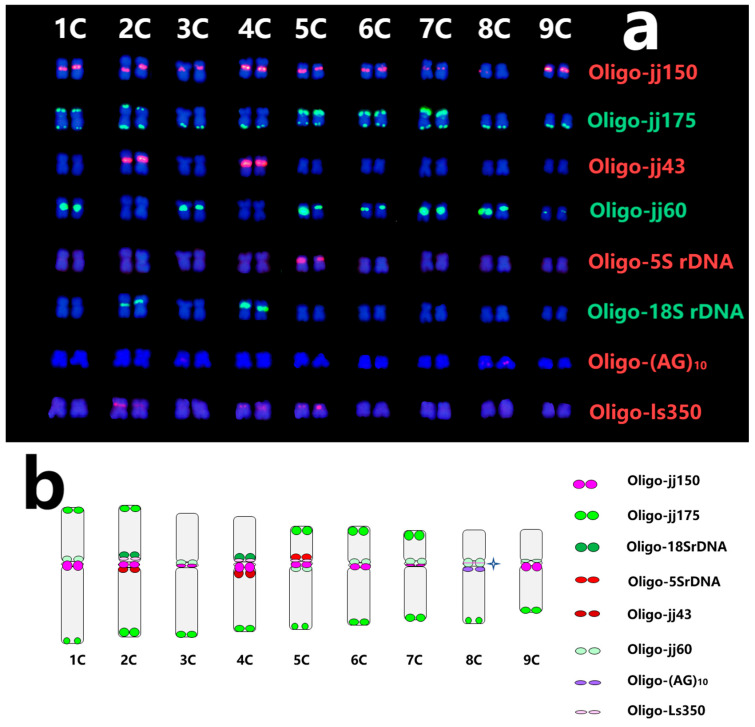
Karyotypes of *Cichorium intybus* cv. Commander with multiple probes (**a**), with the probe list on the right. The ideogram for chromosomes of *Cichorium intybus* cv. Commander showing the distribution of various probes (**b**). The asterisk indicates the polymorphism of the hybridization site of probe Oligo-jj150.

Nine pairs of chromosomes were assigned to the corresponding homology group using ND-FISH patterns and predicted physical locations with multiple probes. The standard karyotypes of *C. intybus* cv. Commander were established (Figure 3a). The hybridization patterns of various probes illustrated the ideogram of 1C to 9C chromosomes of *C. intybus* cv. Commander (Figure 3b). The centromeric and telomeric regions of nine chromosomes show a high density of hybridization sites. Meanwhile, the fluorescence intensity of one probe on different chromosomes was highly variable. For example, probe Oligo-jj60 produced strong hybridization signals on the centromeric region of 5C and 8C, which could not detect any signals on 2C and 4C.

This study also acquired Lettuce’s FISH patterns and ideogram (Figure S3a,b). As a result, the standard karyotypes for *C. intybus* cv. Commander and Lettuce chromosomes were established, providing significant utility for analyzing intraspecific karyotypic variations and revealing genetic diversity within the Cichorieae tribe.

### 2.5. Chromosome Variation in C. intybus cv. Commander

**Figure 4:**
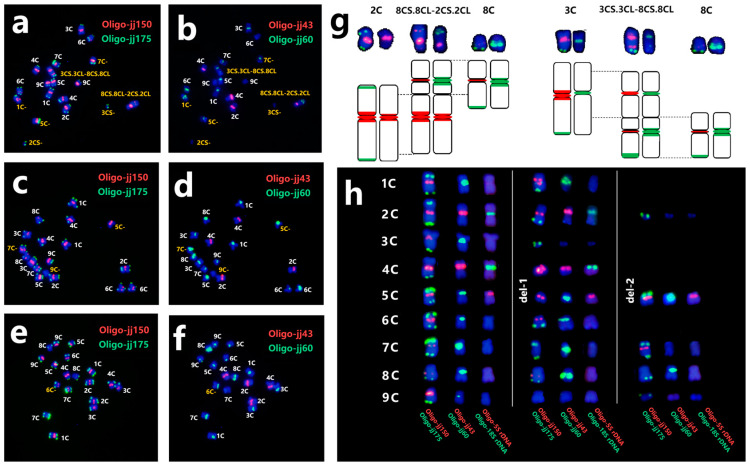
Sequential FISH karyotyping of root-tip cells from lines y64 (**a**,**b**), y61 (**c**,**d**), and y41 (**e**,**f**) revealed chromosomal aberrations. The probes were Oligo-jj150 + Oligo-jj175 (**a**,**c**,**e**), and Oligo-jj43 + Oligo-jj60 (**b**,**d**,**f**). The diagram indicates the rearranged chromosomes 8CS.8CL-2CS.2CL and 3CS.3CL-8CS.8CL (**g**). The probes were Oligo-jj150 + Oligo-jj175 (**right**) and Oligo-jj43 + Oligo-jj60 (**left**). The cut and pasted deletion chromosomes (**h**). The probes were Oligo-jj150 + Oligo-jj175, Oligo-jj43 + Oligo-jj60, and Oligo-5S rDNA + Oligo-18S rDNA from right to left, and the wild-type chromosomes (without deletions) are displayed on the left.

For further cytological investigation on *C. intybus* cv. Commander, 103 plants were subjected to sequential ND-FISH employing several oligo probes to confirm the use of recently produced probes and the standard karyotype. Thirty-three plants (about 32%) have chromosomal abnormalities, according to ND-FISH. As shown in Figure 4a,b, line y64 contains two dicentric translocation chromosomes 8CS.8CL-2CS.2CL and 3CS.3CL-8CS.8CL, as well as five deletions involving 1C, 2C, 3C, 5C, and 7C chromosomes. The ND-FISH patterns for the diagram of the recombination translocations are shown in Figure 4g. Line y53 carried three deletion chromosomes, in which 7C and 5C lost the terminal region with strong Oligo-jj175 signals. Moreover, deletion 9C even lost the Oligo-jj150 hybridization sites on the centromere (Figure 4c,d). Likewise, line y86 also contained deletion 6C with distinct Oligo-jj175 signals on one side (Figure 4e,f). All deletion chromosomes are listed in Figure 4h. Therefore, it is probable that the chromosomes of *C. intybus* cv. Commander has seen significant structural alterations throughout evolution, particularly in the terminal area, and the established karyotype utilizing ND-FISH on Chicory aids in characterizing chromosome translocations and deletions.

### 2.6. Comparative Karyotypic Analysis of Several Cichorium-Related Species

**Figure 5:**
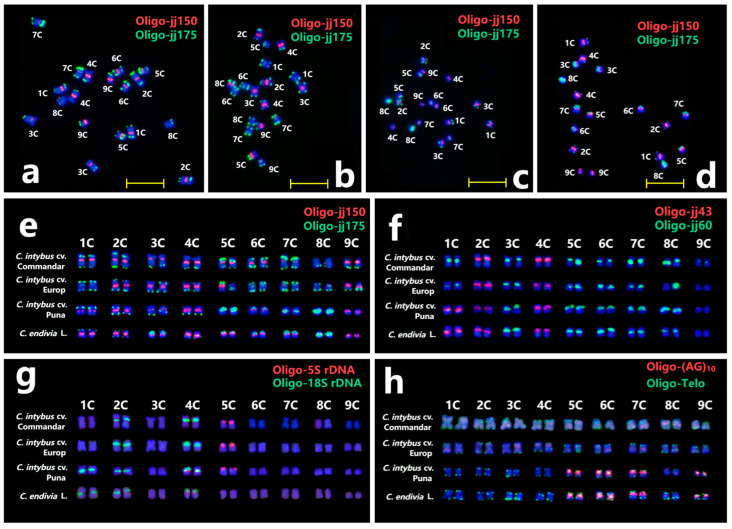
The ND-FISH for karyotyping of *C. intybus* cv. Commander (**a**), *C. intybus* cv. Europe (**b**), *C. intybus* cv. Puna (**c**), and endive (*C. endivia* L.) (**d**) using probes Oligo-jj150 + Oligo-jj175. The cut and pasted chromosomes were characterized using probes Oligo-jj150 + Oligo-jj175 (**e**), Oligo-jj43 + Oligo-jj60 (**f**), Oligo-18S rDNA + Oligo-5S rDNA (**g**), and Oligo-(AG)_10_ +Oligo-telo (**h**).

This study employed sequential ND-FISH on the metaphase cells of endive (*C. endivia* L.) and three Chicory accessions (*C. intybus* cv. Commander, *C. intybus* cv. Puna, and *C. intybus* cv. Europe). The centromere-specific probe Oligo-jj150 demonstrated robust hybridization signals on nine endive and *C. intybus* cv. Puna chromosomal pairs. However, 1C, 6C, and 8C chromosomes in *C. intybus* cv. Europe failed to exhibit any signals. Probe Oligo-jj175 consistently generated clear terminal signals in *C. intybus* cv. Commander and *C. intybus* cv. Europe, but the hybridization sites of Oligo-jj175 gradually accumulated in the subterminal and centromeric regions of *C. intybus* cv. Puna and endive (Figure 5a–e). Similarly, FISH patterns of probes Oligo-jj43 + Oligo-jj60, Oligo-18S rDNA +Oligo-5S rDNA and Oligo-(AG)10 +Oligo-Telo indicated *C. intybus* cv. Puna is more relative to endive (Figure 5f–h). For example, in *C. intybus* cv. Puna and endive, 1C carried an extra 18S rDNA site, and 5C–7C and 9C chromosomes had strong Oligo-(AG)_10_ signals at the centromeric region. However, ND-FISH results revealed the extensive karyotypical diversity of the *Cichorium* genus. As a result, the nomenclature system employing newly designed probes will aid cytogenetic studies in endive and Chicory, enhancing the characterization of evolutionary relationships within the *Cichorium* genus.

## 3. Discussion

The genome sequencing of various types of Chicory and endive has been reported extensively [21,22]. However, a comprehensive examination of the tandem repeat (TR) composition in the aforementioned genomic analysis is lacking. TRs pervading the heterochromatin can be arranged in tandem arrays of thousands of neighboring monomers in the genome. Further, a significant variation in the content and distribution of TRs is commonly observed in the same family. For example, Lang et al. [16] identified 3.06% of the TRs from common wheat (*Triticum aestivum*), in which A, B, and D genomes comprised the average TR content of 2.85%, 3.06%, and 4.03%, respectively. Jiang et al. [23] reported a total length of 447.21 Mb of TR, representing 4.16% of the assembled oat (*Avena sativa*) genome, which is 10.74 Gb in size. The percentage of TRs in the C genome (6.92%) significantly exceeded that of the A genome (2.59%) and the D genome (2.17%). Similarly, the T2T genome of Lettuce, as a relative of Chicory, displayed a comparatively low percentage of satellite sequences (1.00%) and simple repeats (1.11%) [19]. Fan et al. [18] provided chromosome-level reference genomes for four Asteraceae species, revealing that the transposable elements content in endive (18.6%) was nearly three times greater than that in Chicory (6.7%). TR content has significantly expanded in the Asteraceae family during species differentiation, especially in the *Cichorium* genus. In this study, we identified that 7.00% of the genome is composed of TRs for Chicory ASM2352571v1 [18], and found highly accumulated TRs in the 8C (9.17%) and 7C (8.35%) chromosomes. However, only 0.54% of TR content was observed in the Lettuce T2T genome, and the 1C chromosome had the highest proportion of 1.58%. Therefore, further understanding is required regarding the mechanisms through which repetitive elements arise and quickly accumulate in the tribe Cichorieae. Furthermore, predicting TRs may facilitate determining the physical location of chromosome centromeres in Chicory and Lettuce. Most existing plant genome assemblies inadequately represent or completely lack TRs within and near centromeres. Therefore, integrating cytological methods enhances the accuracy and completeness of the assembly in these highly homogeneous and functional TR regions.

Cytogenetical techniques were widely used to study the genetic diversity and polyploidization process in Asteraceae species, including Chrysanthemum species (2n = 18 and 36), *Helianthus annuus* (2n = 34), and *L. sativa* (2n = 2x = 18) [9,24,25]. For previous studies on *Cichorium* species, the primary chromosome number was conserved, and all were 2n = 18 [11]. Bernardes et al. [4] observed the polymorphism of rDNA sites in chromosomes 3 and 8 from different Chicory accessions. The limitations of the karyotyping nomenclature system for Chicory hinder further research on chromosomal rearrangements and species divergence among Chicory accessions and their relatives. This study predicted genome-wide tandem repeats for the *C. intybus* L. genome assembly ASM2352571 v1 and the T2T genome *L. sativa* cv. PKU06, developing nine new oligo probes to characterize individual chromosomes of Chicory and Lettuce. Sequential standard karyotypes of Chicory and Lettuce were established using the combination probes, for example, probes of Oligo-jj150 + Oligo-jj60, Oligo-jj43 + Oligo-jj175, and Oligo-5S rDNA + Oligo-18S rDNA + Oligo-(AG)_10_, which enabled us to produce distinct hybridization signals from chromosomes 1C to 9C in Chicory.

Meanwhile, the ND-FISH patterns of Oligo-jj175 show significant differences with predicted chromosomes, such as chromosomes 2C and 7C (Figure 2b and Figure S1a), which possibly implies that the sequence assembly of the related region in Chicory needs to be continuously updated. Severe cytological variations in the tribe Cichorieae were observed using the FISH procedure, revealing several characterized deletion and translocation chromosomes in Chicory (*C. intybus* cv. Commander) based on the standard karyotype. The new universal chromosome identification system will effectively elucidate genetic diversity, chromosomal rearrangements, and evolutionary relationships among Chicory species through comparative cytogenetic and genomic methodologies.

Recent advancements in technologies such as whole-genome sequencing, bioinformatic analysis, and oligonucleotide probes have significantly broadened the variety of plant chromosome markers and their applications [26]. Certain repetitive sequences specific to particular species can function as genome-specific genetic markers for examining the evolutionary relationships among species [27]. Centromeric repeat sequences are essential to centromere formation, with highly variable copy numbers and rearrangement during speciation and evolution [28,29]. Wang et al. [19] assembled a complete telomere-to-telomere genome of Lettuce, and found that satellites took up a proportion of 16.3% of centromeric repeats. Meanwhile, satellites and *Gypsy* showed colocalization with CENH3, indicating the importance of the centromere function. Most of the centromeric repeats are likely species-specific in plants. For example, the 155 bp satellite repeat CentO in cultivated rice (*Oryza sativa*) was absent in several wild *Oryza* species [30].

Similarly, Quinta demonstrated minimal homology with barley, comparable to the fundamental centromere retrotransposons in wheat [31]. This study predicted the centromeric tandem repeat sequences of Chicory and Lettuce, leading to the development of the centromeric-specific probes Oligo-jj150 and Oligo-ls62. FISH results demonstrated that chromosomes from Chicory and Lettuce showed a single type of hybridization signal from Oligo-jj150 and Oligo-ls62, respectively (Figure S4). Above all, nine pairs of chromosomes in *C. intybus* cv. Puna and *C. endivia* L. displayed strong Oligo-jj150 signals. However, two and three pairs of chromosomes in *C. intybus* cv. Commander and *C. intybus* cv. Europe exhibited slightly weaker or deficient Oligo-jj150 signals (Figure S4a,d). Centromeric repeat sequences in the Cichorieae tribe exhibited high genetic diversity, which drove the rapid evolution during the process of species differentiation.

Polyploidy and genomic instability are crucial for enhancing evolutionary adaptation during speciation [32,33]. The chromosome numbers for the extant Asteraceae family range from n = 2 to 216 [34]. Genome-wide polyploidization and large-scale chromosomal rearrangements may frequently occur during divergence in Asteraceae plants. He et al. [35] compared the ideograms within the Artemisia genus, demonstrating significant genetic diversity and heterozygosity frequency through ND-FISH methodologies. Bernardes et al. [4] detected heteromorphism related to the distribution of the rDNA sites on chromosome pairs 1 and 3 in two Chicory accessions. In our study, *C. intybus* cv. Europe contained two Oligo-5S rDNA sites on chromosome 5C, which exhibited high heterozygosity with entirely different hybridization patterns of probes Oligo-jj175 + Oligo-jj150 (Figure S5e–g). Similarly, ND-FISH of probe Oligo-Telo indicated the presence of signals at chromosomal regions other than the telomeres in *C. intybus* cv. Commander and *C. intybus* cv. Europe (Figure 5h), which may be a consequence of telomere repeat duplications elsewhere or chromosome breakage and telomere fusion events with other chromosomes [36,37]. Moreover, the distribution of centromere-specific repeats suggests that the average copy number of Oligo-jj150 among 1C to 9C is 3013. However, 7C and 8C displayed the lowest copy numbers, approximately 351 and 587, respectively, as corroborated by the weakly detectable signals of Oligo-jj150 in both 7C and 8C. Meanwhile, the polymorphic FISH signals of Oligo-jj150 were also observed in chromosome 8C (Figure S5a–d). Extensive chromosome rearrangements may have occurred in 7C and 8C, causing losses of tandem repeat sequences in the centromeric region.

Moreover, the hybridization signals of probes Oligo-jj60 were observed in the centromeric region of Chicory chromosomes, except for 2C and 4C. On the other hand, probe Oligo-jj43 generated strong signals exclusively in the centromeric regions of the 2C and 4C chromosomes, suggesting that the centromeric repeats across different chromosomes of Chicory evolve independently. Thus, the centromere-specific oligo probes of Chicory developed in our study could provide insights into the mechanism of high genetic diversity, heterozygosity, and differential centromeric repeat sequence variation in *C. intybus* L. and its relatives. Future research will expand this study to include various diploid and tetraploid species to measure the extent of intra- and interspecific variation. The high sequence similarity among homoeologous regions in polyploid genomes can be distinguished, providing a foundation for future polyploid genome assemblies.

## 4. Materials and Methods

### 4.1. Plant Materials

The Chicory (*C. intybus* L.) accessions (including *C. intybus* cv. Commander, *C. intybus* cv. Europe, *C. intybus* cv. Puna), and endive (*C. endivia* L.) were provided by Forestry and Bamboo Technology Innovation Industry Research Institute, Leshan Normal University. Lettuce (*Lactuca sativa* L.) and Stem Lettuce (*Lactuca sativa* L.) were obtained from the Chinese Crop Germplasm Information Network. In this analysis, 889 plant seeds were combined with mitotic plates from 103, 67, 85, 103, 144, and 74 individuals analyzed for the cultivars *C. intybus* cv. Commander, *C. intybus* cv. Europe, *C. intybus* cv. Puna, *C. endivia* L., Lettuce, and Stem Lettuce. The selection of mitotic plates was based on the quality of the metaphase spreads, ensuring they were free from contamination and contained many intact metaphase cells.

### 4.2. Bioinformatic Analysis of Tandem Repeats in Chicory and Lettuce Genomes

The genome sequences, including Chicory (ASM2352571v1) [19] and Lettuce telomere-to-telomere (T2T) genome of *L. sativa* cv. PKU06) [20] were downloaded to predict tandem repeats using the TRF software [38]. Four developed probes (Oligo-Telo, Oligo-(A)20, Oligo-5S rDNA, and Oligo-18S rDNA) alongside nine newly synthesized oligo probes derived from Chicory genomes (Oligo-jj43, Oligo-jj60, Oligo-jj150 and Oligo-jj175) and Lettuce genomes (Oligo-ls40, Oligo-ls62, Oligo-ls350, Oligo-ls368 and Oligo-ls491) were employed for ND-FISH. All probes are detailed in Table 2. The hybridization locus and copy number of tandem repeats were predicted using the web-based server B2DSC with default parameters (http://mcgb.uestc.edu.cn/b2dsc (accessed on 10 May 2024)) [16].

### 4.3. Chromosome Preparation and FISH Analysis

Root tips from germinated seeds were collected and treated by nitrous oxide and sequential enzymatic digestion, and the preparation of mitotic metaphase chromosome followed the description of Han et al. [41]. All oligonucleotide probes were either 5′ end-labeled with 6-carboxyfluorescein (6-FAM) for green or 6-carboxytetramethylrhodamine (Tamra) for red signals. The protocol of non-denaturing FISH (ND-FISH) using synthesized probes was described by Fu et al. [42]. The hybridization conditions were optimized to achieve an equilibrium between signal intensity and background noise, using a hybridization temperature of 42 °C for 50 min. Photomicrographs of the FISH chromosomes were obtained using an Olympus BX-53 microscope fitted with a DP-70 CCD camera (Shinjuku Monolith, Shinjuku-ku, Japan).

## 5. Conclusions

In conclusion, the physical mapping of labeled probes derived from the genome sequences demonstrates that the consensus karyotype based on ND-FISH can be an effective alternative to traditional cytological methods for identifying genomic variations in the genus *Cichorium*. The present study indicates that ND-FISH utilizing nine newly developed oligo probes can produce a high-resolution and informative cytogenetic map of genomic regions in Chicory and Lettuce. The standard karyotypes with numerous probes show the intraspecific karyotypic differences in *Cichorium* species. Meanwhile, the accurate characterization of centromere-specific tandem repeats will reveal new information on chromosome evolution and genetic diversity in the Asteraceae family.

## Figures and Tables

**Table 1:** The predicted total TRs in the chromosomes of the Chicory and Lettuce genomes using TRF.

Chromosome	TR Total Length (bp)	Chromosome Length (bp)	TR Content (%)	Chromosome	TR Total Length (bp)	Chromosome Length (bp)	TR Content (%)
1C	10,840,173	161,702,566	6.7	1L	4,077,116	258,130,948	1.58
2C	9,312,424	159,989,680	5.82	2L	629,559	238,535,665	0.26
3C	9,318,956	157,584,665	5.91	3L	598,133	320,657,391	0.19
4C	9,394,122	142,005,817	6.61	4L	2,718,271	409,415,085	0.66
5C	10,118,225	126,222,701	8.02	5L	2,281,982	373,650,124	0.61
6C	6,982,906	121,338,966	5.75	6L	351,418	207,794,748	0.17
7C	9,970,721	119,386,547	8.35	7L	641,683	210,595,814	0.3
8C	10,811,047	117,937,712	9.17	8L	1,531,263	345,107,305	0.44
9C	7,606,651	98,217,271	7.74	9L	1,174,833	229,575,853	0.51
Total	84,355,225	1,204,385,925	7.00	Total	14,004,258	2,593,462,933	0.54

**Table 2:** The sequences of oligo probes for Chicory chromosome identification by ND-FISH.

Oligo Probes	Sequences	Reference
Oligo-(AG)_10_	AGAGAGAGAGAGAGAGAGAG	[39]
Oligo-Telo	TTTAGGGTTTAGGGTTTAGGG	[40]
Oligo-5S rDNA	TCAGAACTCCGAAGTTAAGCGTGCTTGGGCGAGAGTAGTAC	[16]
Oligo-18S rDNA	GGGCAAGTCTGGTGCCAGCAGCCGCGGT	[16]
Oligo-jj43	GACCCATGGACGACCAGGTGAACAAGGCACCGTGCCATACACA	This study
Oligo-jj60	ACCCTAATACATCATATGAAAACTAAAAAACCCTAATCCATCATATG	This study
Oligo-jj150	GATGGGTTATGGTCAACCAGGTAGGGTTGTGATGGGTTATGGTCAACC	This study
Oligo-jj175	TACACCAGAAATGTTTAAAATGATCACAAAGTGTAATTATAGGTAATTTAT	This study
Oligo-ls40	CACAACCAACACCCATACATGACCTAATGAGGTCCAAACC	This study
Oligo-ls62	AATTTAGCAAAGCTTTGATGAGCATTGTTTAATTCTTGAGTTCCAC	This study
Oligo-ls350	GTACGCGGGGCGTGGGACCCACGTGACCGATCGGCTCCGTTCC	This study
Oligo-ls368	AGAATGAGAACATAATAATGTTGATATAAAGTTGCTAA	This study
Oligo-ls491	ATCATGAGTTCCTTACGCAAATATCGCCATAATTGTTGT	This study

## Data Availability

The data that support the findings of this study are included in this published article and the Supplementary Materials file.

## Supplementary Materials

The following supporting information can be downloaded at https://www.mdpi.com/article/10.3390/plants13223135/s1: Figure S1: The predicted physical distribution of nine newly developed oligo probes in Chicory (a) and Lettuce (b). The probes used are listed on the bottom right. Figure S2: ND-FISH using multiple probes in Chicory. The probes for FISH were Oligo-jj150 (red) + Oligo-jj175 (green) (a,c); Oligo-(AG)_10_ (red) + Oligo-Telo (green) (b); Oligo-ls350 (red) (d). Figure S3: Karyotypes of Lettuce with multiple probes (a), with the probe list on the right. The ideogram for chromosomes of Lettuce shows the distribution of numerous probes (b). Figure S4: Karyotyping of mitotic metaphase of *C. intybus* cv. Commander (a), *C. intybus* cv. Puna (b), *C. endivia* L. (c), *C. intybus* cv. Europe (d), Lettuce (*Lactuca sativa* L.) (e), and stem Lettuce (*Lactuca sativa* L.) (f) using probes Oligo-jj150 (red) + Oligo-ls62 (green). Asterisks indicate the chromosomes with weak or no Oligo-jj150 signals. Figure S5: Karyotyping of mitotic metaphase of *C. intybus* cv. Commander (a), *C. intybus* cv. Puna (b), *C. endivia* L. (c), *C. intybus* cv. Europe (d), Lettuce (*Lactuca sativa* L.) (e), and Stem Lettuce (*Lactuca sativa* L.) (f) using probes Oligo-jj150 (red) + Oligo-ls62 (green). The asterisk indicates the chromosomes with weak or no Oligo-jj150 signals. Table S1: Distribution and copy number prediction of Oligo-18S rDNA (left) and Oligo-5S rDNA (right) in Chicory (2n = 18, CC) and Lettuce (2n = 18, LL) genomes. Table S2: Distribution and copy number prediction of centromere-specific probes Oligo-jj150 (left) and Oligo-ls62 (right) in Chicory (2n = 18, CC) and Lettuce (2n = 18, LL) genomes.
