# Supplementary material for: ND-FISH with New Oligo Probes for Chromosome Identification of Cichorium intybus Revealing Karyotypic Variation and Divergence of Asteraceae Species
Source: Plants (Basel). 2024 Nov 7;13(22):3135. doi: 10.3390/plants13223135 (PMC11598091; doi:10.3390/plants13223135)
Supplement: Supplementary file 1 [file plants-13-03135-s001.zip › plants-3265572-supplementary.pdf]

Suplementary Information

ND-FISH with New Oligo Probes for Chromosomes Identification of *Cichorium intybus* Revealing karyotypic variation and divergence of Asteraceae species

Meiling Chen [1,2,†], Chengzhi Jiang [2,†], Doudou Huang [2], Zhiqiang Zheng [2], Wenzhuo Yang [2], Guangrong Li [2], Chun Fu [1,3], Hong Liao [1,3], Wencong Long [1,3], Zujun Yang [2,*] and Yaojun Yang [1,3,*]

[1] Forestry and Bamboo Technology Innovation Industry Research Institute, Leshan Normal University,
Leshan 614000, China; cmelia123@163.com (M.C.); fuchun421@aliyun.com (C.F.); 18608332688@163.com (H.L.);
longwencong@163.com (W.L.)

[2] School of Life Science and Technology, University of Electronic Science and Technology of China,
Chengdu 610054, China; 202311140626@std.uestc.edu.cn (C.J.); 202221140532@std.uestc.edu.cn (D.H.); 202221140503@std.uestc.edu.cn (Z.Z.); 2022140902006@std.uestc.edu.cn (W.Y.); ligr28@uestc.edu.cn (G.L.)

[3] Key Laboratory of Sichuan Province for Bamboo Pests Control and Resource Development, Leshan Normal University, Leshan 614000, China

* Correspondence: yangzujun@uestc.edu.cn (Z.Y.); rsyyj@126.com (Y.Y.)

† These authors contributed equally to this work.

Supplementary materials

Table S1. Distribution and copy number prediction of Oligo-18S rDNA (left) and Oligo-5S rDNA (right) in chicory (2n=18, CC) and lettuce (2n=18, LL) genome.

Chromosomes	Position (Mb)	Copy number	Chromosomes	Position (Mb)	Copy number
2C	63-64	110	5C	96-99	9150
4C	87-90	209	1L	201-202	1881
1L	254-258	337	1L	205	430
8L	339-345	531			

Table S2. Distribution and copy number prediction of centromere specific probes Oligo-jj150 (left) and Oligo-ls62 (right) in Chicory (2n=18, CC) and Lettuce (2n=18, LL) genome.

Oligo-jj150			Oligo-ls62		
Chromosome s	Position (Mb)	Copy number	Chromosome s	Position (Mb)	Copy number
1C	90-95	2482	1L	181-183	3823
2C	78-81	2309	2L	89-91	8035
3C	96-101	3399	3L	219	333
4C	77-81	4164	5L	115-120	3961
5C	86-89	2100	6L	108-109	761
6C	75-82	2476	7L	113-116	4141
9C	62-67	885	8L	261-262	12195
			9L	132-135	4203

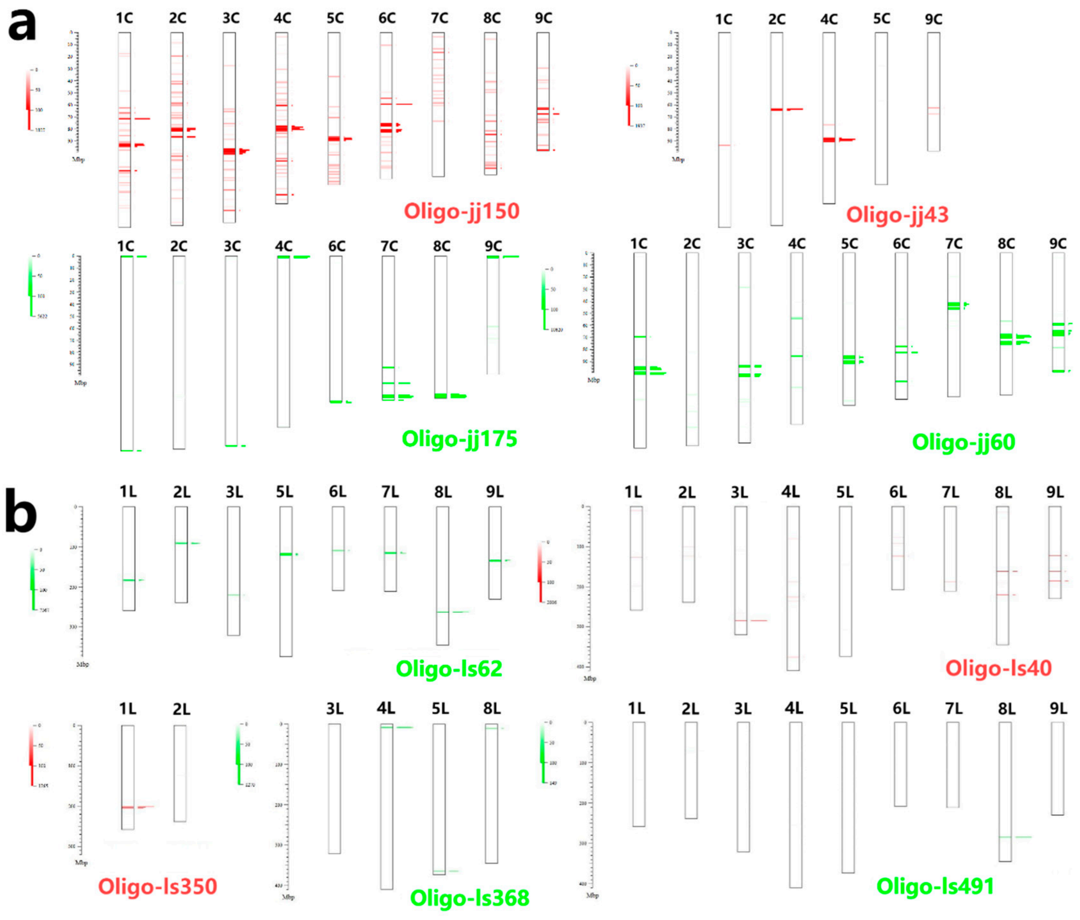

Figure S1. The predicted physical distribution of nine newly developed Oligo probes in Chicory (a) and Lettuce (b). The probes used were marked on the bottom right.

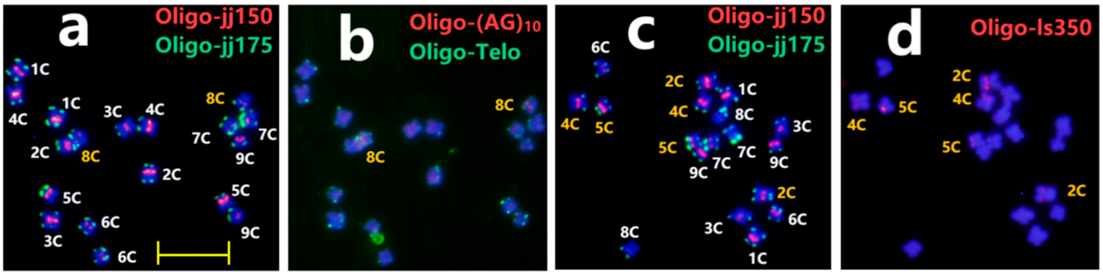

Figure S2. ND-FISH using multiple probes in chicory. The probes for FISH were: Oligo-jj150 (red) + Oligo-jj175 (green) (a, c); Oligo-$(AG)_{10}$ (red) + Oligo- Telo (green) (b); Oligo-ls350 (red) (d).

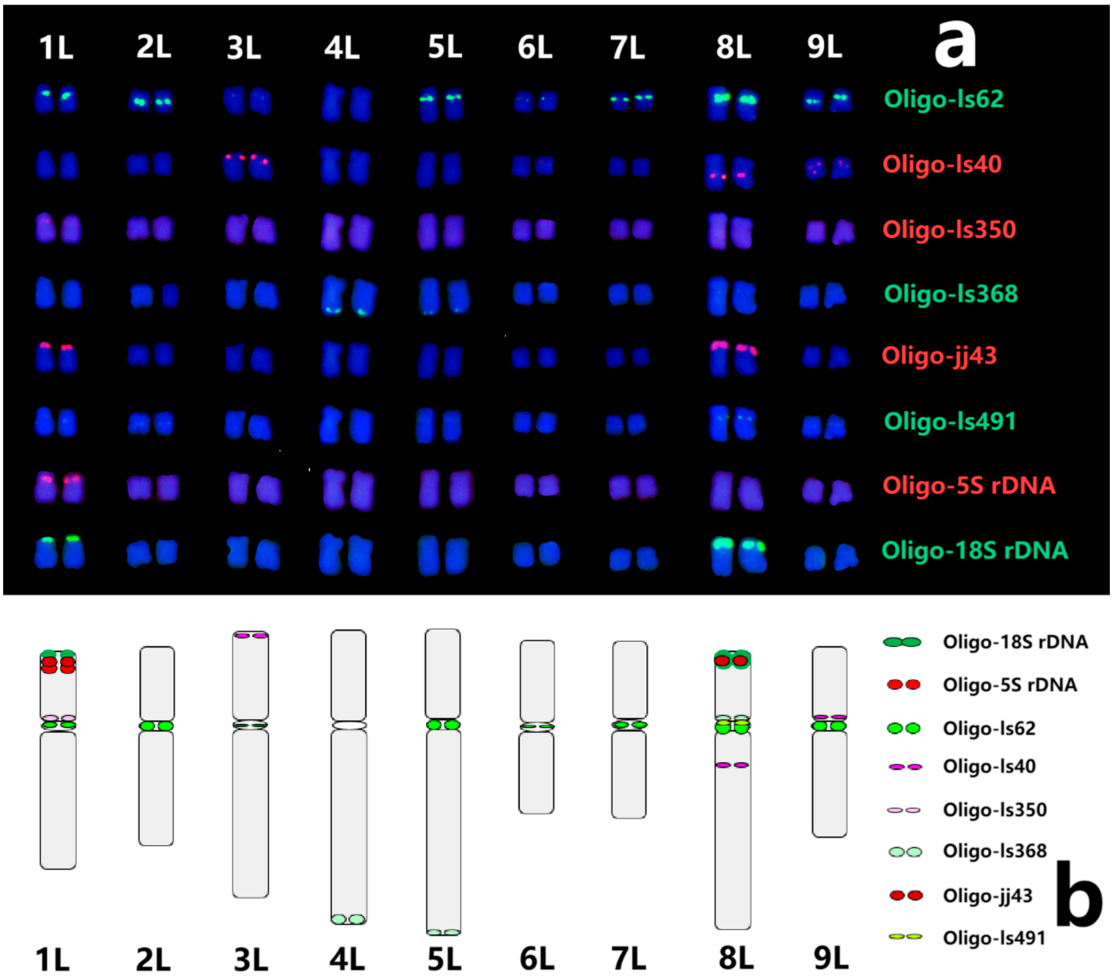

Figure S3. Karyotypes of Lettuce with multiple probes (a), the probes list on the right. The ideogram for chromosomes of Lettuce shows the distribution of numerous probes (b).

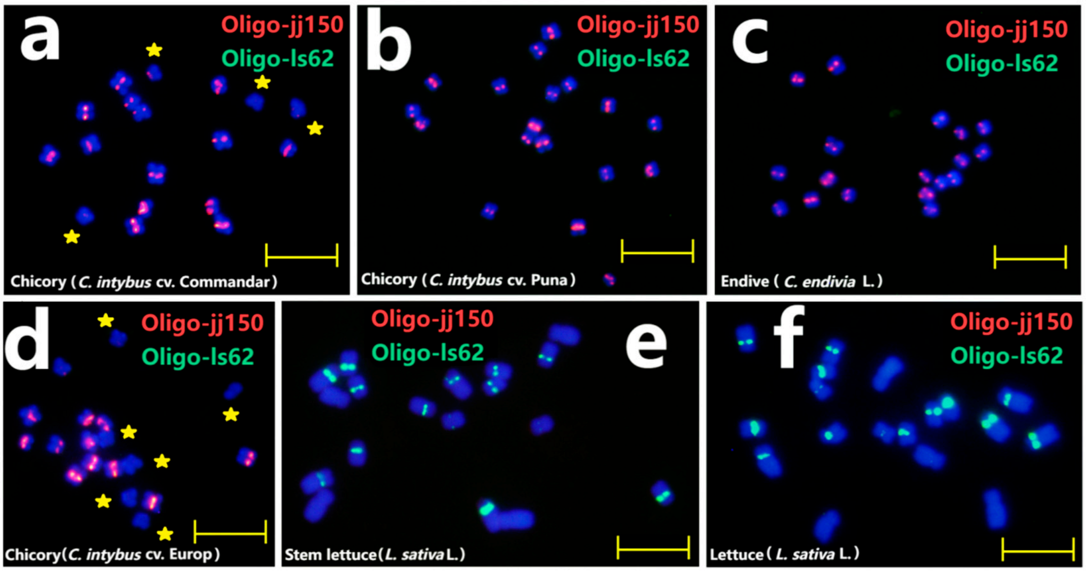

Figure S4. Karyotyping of mitotic metaphase of *C. intybus* cv. Commander (a), *C. intybus* cv. Puna (b), *C. endivia* L. (c), *C. intybus* cv. Europe (d), Lettuce (*Lactuca sativa* L.) (e) and stem Lettuce (*Lactuca sativa* L.) (f) using probes Oligo-jj150 (red) + Oligo-ls62 (green). Asterisk indicated the chromosomes with weak or no Oligo-jj150 signals.

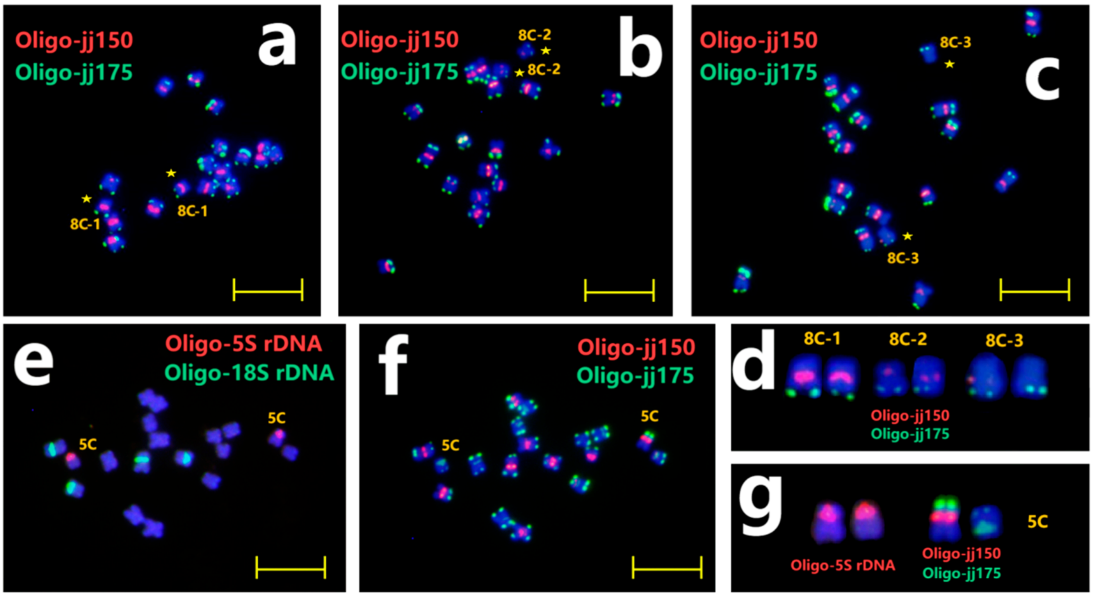

Figure S5. Karyotyping of mitotic metaphase of *C. intybus* cv. Commander (a), *C. intybus* cv. Puna (b), *C. endivia* L. (c), *C. intybus* cv. Europe (d), Lettuce (*Lactuca sativa* L.) (e) and stem lettuce (*Lactuca sativa* L.) (f) using probes Oligo-jj150 (red) + Oligo-ls62 (green). The asterisk indicated the chromosomes with weak or no Oligo-jj150 signals.
